# Adolescents’ Social Media Use and Their Voluntary Garbage Sorting Intention: A Sequential Mediation Model

**DOI:** 10.3390/ijerph18158119

**Published:** 2021-07-31

**Authors:** Pengya Ai, Wu Li, Wuyue Yang

**Affiliations:** School of Media and Communication, Shanghai Jiao Tong University, No. 800, Dongchuan Road, Minhang District, Shanghai 200240, China; aipengya1997@163.com (P.A.); summercury@sjtu.edu.cn (W.Y.)

**Keywords:** pro-environmental behavior, garbage sorting, adolescents, social media use

## Abstract

Encouraging adolescents to sort garbage is vital for the sustainable development of the ecological environment. This study investigates the relationship between adolescents’ social media use and their garbage sorting intention. A survey, in both online and paper-based versions, was distributed in 2020 in Shanghai and a total number of 605 valid responses were obtained. This study supports the proposed sequential mediation model, indicating that adolescents’ social media use increased their garbage sorting intention via the serial mediation effect of objective and subjective knowledge and the perceived importance of garbage sorting. The current study and its findings provide important insights into the link between adolescents’ social media use and their garbage sorting intention, particularly its underlying mechanism, by distinguishing knowledge into two specific types and introducing perceived importance into the conceptual model. Practical implications for improving adolescents’ intention to sort garbage are also discussed.

## 1. Introduction

Domestic garbage in China has reached extremely large amounts, and the number in 2019 has exceeded 242 million tons [1]. Such high amounts of garbage have caused prominent environmental hazards; therefore, safe disposal is crucial. Although many developed countries have implemented garbage sorting methods with a mature track record, China has only recently begun to pilot and promote garbage sorting in Shanghai [2]. Therefore, it is imperative for those involved in protecting the environment to encourage garbage sorting behavior, a type of pro-environmental behavior. Studies have indicated that adolescents have less interest in and fewer concerns about environmental issues and sustainable development [3]. Therefore, more academic attention should be paid to this specific age group when studying how to encourage garbage sorting intention [3].

Media can be used to encourage pro-environmental behavior [4,5]. Social media, which differs from traditional media in its combination of mass communication and interpersonal communication, has developed rapidly with increasing penetration into people’s daily consumption of media. For example, the number of WeChat users reached 1.15 billion in 2019 [6]. Social media has also become a compelling and integral part of adolescents’ daily lives worldwide [7]. However, despite the significance of social media for adolescents, the association between adolescents’ social media use and pro-environmental behavior has rarely been studied.

To fill this research gap, the current study intends to explore the potential effects of social media on encouraging adolescents’ willingness to participate in garbage sorting. Moreover, this study seeks to examine the mediation effects of objective knowledge, subjective knowledge, and perceived importance of sorting garbage. Using the sequential mediation model, this study provides insights into the understanding of the mechanisms behind the effects of social media on adolescents’ voluntary garbage sorting intention, and offers valuable references for practitioners to improve adolescents’ intention to sort garbage as well.

## 2. Literature Review

### 2.1. Social Media Use and Garbage Sorting Intention

Pro-environmental behavior refers to the type of environmental action that an individual consciously chooses to minimize the negative impact of their activities on the natural and constructed world, and is directly associated with environmental improvement [8]. Pro-environmental behavior is seen by many scholars as a multi-dimensional construct. For example, Larson et al. identified pro-environmental behavior with four domains, including conservation lifestyle behaviors, social environmentalism, environmental citizenship, and land stewardship [9]. Meanwhile, garbage sorting behavior is regarded as a specific type of pro-environmental behavior. 

According to Social Cognitive Theory (SCT), people can learn their behavior by observing the behavior of others and the outcomes of their actions, either in real life or from media [10]. As an important channel for learning social norms [11,12], media has the function of social integration, which requires people to follow social rules [13]. Practically, media has been employed in environmental advertisement campaigns, environmental policy promotion, and informing the public of environmental knowledge. Based on SCT, exposure to garbage sorting issues on social media offers individuals opportunities to observe actual garbage sorting behaviors. As such, individuals will imitate this behavior by consuming such content in media. 

Empirical studies indicate that media use positively predicts pro-environmental behavior. For instance, Lee found that media attention on environmental issues and the appeal for environmental protection promote the public to value environmental participation and enhance their intention to participate in such actions [14]. Huang revealed that exposure to global warming media coverage has direct positive impacts on three forms of pro-environmental behavior (i.e., accommodating, promotional and proactive behavior) [5]. Apart from mass media, internet or social media have also been found to be associated with pro-environmental behavior. Han and Xu found that social media use can strengthen individuals’ pro-environmental behavior through interpersonal communication [15]. Furthermore, Ma and Zhu revealed that internet use positively influences Chinese residents’ willingness to participate in garbage sorting [16]. While none of these studies focused on the garbage sorting intention of adolescents, according to their findings, we can assume that adolescent social media use may also positively affect their garbage sorting behavior.

Due to the compulsory policy in Shanghai, citizens are required to sort their garbage. According to Azjen, behavioral intention can be treated as a proxy for actual behavior [17]. Consequently, this study employs adolescents’ intention to voluntarily sort garbage as a dependent variable to avoid the potential condition that the measurement of garbage sorting behavior lacks sufficient variation. This study suggests that social media use can positively influence adolescents’ voluntary garbage sorting intention, and thus proposes the following hypothesis:

**Hypothesis** **1** **(H1).** *Social media use is positively associated with adolescent voluntary garbage sorting intention.*

### 2.2. Perceived Importance

Regarding ethical issues, perceived importance is a variable that precedes moral behavior intention. The more perceived importance an issue has, the higher the probability that an individual will make morally desirable decisions and engage in morally desirable behaviors [18]. Robin et al. defined the perceived importance of an ethical issue to an individual as “the perceived personal relevance or importance of an ethical issue to an individual [19]”. Empirical studies have revealed perceived importance as a significant predictor of ethical behavior. For example, Culiberg and Mihelič found that the higher the perceived importance of peer reporting is in an ethical decision-making process, the higher the student’s intention to engage in peer reporting themselves [20]. To a certain degree, pro-environmental behavior is a moral or ethical issue [21]; therefore, an individual’s perceived importance of environmental issues should associate with pro-environmental behavior. 

Based on the General Learning Model, media—a situational variable—can affect an individual’s behavior by firstly changing their internal state [22]. Media is an important channel through which to inform individuals about environmental risks and consequently raise their perception of risk on environmental issues [23]. Given that numerous environmental campaigns have been conducted on social media, it is clear that social media is capable of eliciting environmental awareness on a large scale and at a high speed [24]. Thus, as individuals are more exposed to environmental issues and campaigns on social media and become more engaged in discussions on those issues, they are more likely to perceive the significance of the environmental problems we are currently facing.

Consequently, this study posits that the more individuals are exposed to and discuss garbage sorting issues on social media, the more likely they will perceive garbage sorting as important for the environment, and as such, they will be more likely to participate in garbage sorting. Therefore, this study proposes the following hypotheses:

**Hypothesis** **2** **(H2).** *Perceived importance is positively associated with adolescents’ voluntary garbage sorting intention.*

**Hypothesis** **3** **(H3).** *Social media use is positively associated with adolescents’ perceived importance of garbage sorting.*

### 2.3. Objective Knowledge and Subjective Knowledge

Environmental knowledge is defined as a person’s general understanding of the facts, concepts, and relationships associated with the natural environment and its ecosystems [25]. Brucks distinguished two forms of knowledge: objective knowledge and subjective knowledge [26]. Objective knowledge, also known as factual knowledge, is an individual’s understanding of factually accurate information 26]. Whereas subjective knowledge is an individual’s perception of the amount of knowledge they have, namely what an individual perceives but does not technically represent how much they actually know 26]. 

Objective knowledge is an outcome variable that has gained wide interest in science communication [27]. However, some studies have revealed that subjective knowledge is a more important predictor of individual behaviors than objective knowledge [28]. Hence, this study incorporates both forms of knowledge to investigate their roles in adolescents’ garbage sorting behaviors. Furthermore, the literature also indicates a potential relationship between factual and subjective knowledge. For example, Pieniak et al. found that objective knowledge positively predicted subjective knowledge in organic vegetables consumption [28]. A meta-analysis in consumer research also found that there exists the relationship between objective knowledge and subjective knowledge in various contexts including products, non-products, public goods, and private goods [29].

Some theories have suggested that mass media use is closely related to an individual’s knowledge acquisition. For instance, Uses and Gratifications Theory claims that an important function of media, is to satisfy people’s cognitive needs [30]. The Cognitive Mediation Model indicates that exposure to media content is associated with elaborative information processing, which is a precondition of gaining objective knowledge [31]. At the same time, the relationship between mass media use and knowledge acquisition has been supported by various empirical studies. Yang et al. found that attention to newspaper and TV programs is positively associated with an individual’s factual knowledge and perceived familiarity (a concept similar to subjective knowledge) with nanotechnology [32]. Similarly, Ho et al. found that news attention positively predicts both factual and subjective knowledge on science and technology [33].

While mass media has been proved to be used by individuals to get information and knowledge, interpersonal communication is also an essential tool for them to seek information [34]. Beyond that, interpersonal communication can also enhance individuals’ learning processes and help them to gain knowledge [35]. Therefore, the use of social media, which combines the functions of mass media and interpersonal communication, should positively predict one’s knowledge acquisition. That is, the more adolescents use social media to obtain garbage sorting information and engage in interpersonal interactions on social media, the more objective knowledge they will gain. Meanwhile, the more objective knowledge adolescents possess, the more they will think they know, which will increase their subjective knowledge. As a result, this study proposes the following hypotheses:

**Hypothesis** **4** **(H4).** *Adolescents’ possession of objective knowledge is positively associated with their possession of subjective knowledge on garbage sorting.*

**Hypothesis** **5** **(H5).** *Social media use is positively adolescents’ with adolescent possession of (a) objective knowledge and (b) subjective knowledge on garbage sorting.*

### 2.4. Knowledge, Perceived Importance, and Behavioral Intention

An individual’s environmental knowledge is an indication of an individual’s awareness and knowledge about environmental problems [36], which is treated as the cognitive component of environmental awareness [37]. Given the definition of environmental knowledge, the more knowledge an individual possesses, the more they should know about environmental problems, the relationship between human activities and the natural environment, and the possible consequences of the continuous deterioration of the environment. Therefore, people with more environmental knowledge are inclined to have higher perceived importance of garbage sorting and are more willing to engage in actual behaviors as well. Moreover, as the knowledge an individual possesses increases, they will know more about how to perform pro-environmental behavior and have better control over these actions. As a result, their behavior intention will increase according to the Theory of Planned Behavior [4,17]. 

Environmental knowledge has gained widespread attention in pro-environmental behavior studies and is widely regarded as an important personal factor that impacts individuals’ environmental attitudes and pro-environmental behaviors [38,39,40,41,42]. When considering the two forms of knowledge separately, both objective knowledge and subjective knowledge are found to be associated with pro-environmental behaviors. For example, Ellen revealed that these two types of knowledge positively predict people’s such pro-environmental behaviors as recycling and source reduction [43]. A meta-review has also shown that correct knowledge (i.e., objective knowledge) positively predicts behavior, and so does self-reported knowledge (i.e., subjective knowledge) yet with a more predicting power [38]. Furthermore, Pieniak et al. found that objective knowledge is indirectly associated with organic vegetable consumption through the mediation of subjective knowledge [28]. 

Within the context of garbage sorting, this study reasons that as adolescents acquire objective knowledge on garbage sorting through social media, they will perceive that their knowledge on this subject is increasing. As both objective and subjective knowledge increase, they will be more likely to be aware of the significance and value of garbage sorting and thus voluntarily engage in garbage sorting even if there are no compulsory requirements. Consequently, this study posits the following hypotheses:

**Hypothesis** **6** **(H6).** *Adolescents’ (a) objective knowledge and (b) subjective knowledge are positively associated with their perceived importance of garbage sorting*

**Hypothesis** **7** **(H7).** *Adolescents’ (a) objective knowledge and (b) subjective knowledge are positively associated with their voluntary garbage sorting intention.*

**Figure 1:**
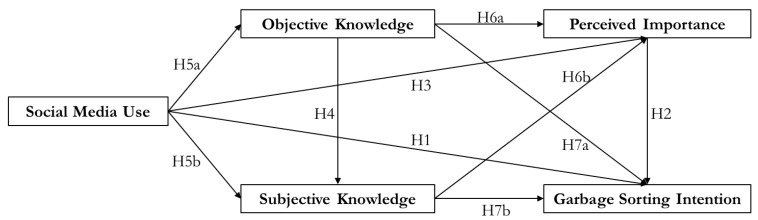
Proposed research model.

The proposed research model is presented below (Figure 1).

## 3. Materials and Methods

### 3.1. Sample

A survey, in both online and paper-based versions, was conducted in 2020 in Shanghai. The study was conducted in accordance with the Declaration of Helsinki, and the protocol was approved by the Institutional Review Board of Shanghai Jiao Tong University (No. H20200381). The research procedures are described as follows. Firstly, we divided Shanghai into three areas (i.e., Pudong new area, urban Puxi area, and rural Puxi area) due to different conditions of development. From each area, we randomly selected one junior middle school and one senior middle school. Secondly, we contacted the principal of each middle school for their permission to carry out the survey. After receiving their permission, we randomly selected classes from each middle school, and the teacher of each class distributed the questionnaire during break time. Informed consent was obtained from all subjects involved in the study. Due to the COVID-19 pandemic, the whole process lasted three months, and 628 adolescents participated in the survey. After data cleaning (dropping all cases with missing values), a total number of 605 valid responses were kept for data analysis. Of the respondents, 53.7% were female, and 46.3% were male; 60.5% were junior high school students, and the rest were senior high students. The age range of the respondents is 11 to 18 years (M = 14.226, SD = 1.978).

### 3.2. Measurement

**Social media use (SMU).** Social media use was measured by two items regarding the two main functions of social media use [44]. With a 5-point Likert scale (1 = never, 5 = always), participants were asked to indicate how often they gain information on or discuss with others about garbage sorting issues using social media (Cronbach’s alpha = 0.814).

**Objective knowledge (OK).** Aligned with the objective knowledge measurement of previous studies [27,33], this study measured objective knowledge of garbage sorting with 10 dichotomous questions adapted from the most recent garbage sorting policies and approaches in Shanghai [45]. Participants answered if each of the statements were “true”, “false”, or they “don’t know”. The statements included “Waste fabrics (such as plush toys, bedsheets) are recyclable (T)”, “Wet garbage is treated using incineration (F)”, “Catering service providers should not actively provide consumers with disposable chopsticks, spoons, and other tableware (T)”, etc. Participants would receive one point for each correct answer, while zero points were given if the answer was incorrect or “don’t know”. A final score for each participant was calculated by the sum of the 10 questions and represented their objective knowledge.

**Subjective knowledge (SK).** Subjective knowledge was measured as the participants’ current perceived levels of knowledge on garbage sorting [33]. Participants would indicate how much they know about (a) the criteria and rules of garbage sorting, (b) the garbage sorting policies, (c) the follow-up garbage processing methods, and (d) the possible outcomes of improper garbage disposal on a 5-point Likert scale (1 = not at all, 5 = very much). The four items yielded a Cronbach’s alpha of 0.847, indicating high reliability.

**Perceived importance (PI).** Perceived importance of garbage sorting was measured using five 5-point items (1 = strongly disagree, 5 = strongly agree) [18]. Participants were asked to indicate their agreement of five statements including; (a) “It’s important to sort garbage”, (b) “It’s essential to classify garbage”, (c) “Garbage sorting is of great value”, (d) “Not sorting garbage will bring about very harmful outcomes”, and (e) “If we don’t sort garbage, we will soon face very severe environmental problems” (Cronbach’s alpha = 0.961).

**Garbage sorting intention (GSI).** Garbage sorting intention was measured on a 5-point Likert scale (1 = not at all, 5 = very much) [18]. Participants were asked to indicate how willing they are to sort garbage if garbage sorting is not mandatory.

Gender and age were used as controlled variables. The highest form of formal education the respondents’ parents received (M = 15.436, SD = 1.988) were also controlled. 

Descriptive statistics and correlation coefficients of key variables are listed in Table 1.

## 4. Results

PROCESS SPSS macro (Hayes, A.F., Lawrence, KS, USA) was employed to examine the moderated mediation model (Model 6) [46]. The regression results (see Table 2) found that social media use positively predicts adolescents’ objective knowledge possession (*β* = 0.2397, *p* = 0.0006, 95% CI = [0.1037, 0.3757]) and their subjective knowledge possession (*β* = 0.2036, *p* < 0.0001, 95% CI = [0.1475, 0.2596]). However, on 0.05 significance level, social media use does not predict the perceived importance of garbage sorting (*β* = 0.0401, *p* = 0.2154, 95% CI = [−0.0234, 0.1036]) and voluntary behavioral intention (*β* = 0.0741, *p* = 0.0633, 95% CI = [−0.0041, 0.1523]). These results support H5a and H5b, while H1 and H3 are not supported.

Objective knowledge positively predicts adolescents’ subjective knowledge (*β* = 0.0710, *p* < 0.0001, 95% CI = [0.0382, 0.1037]) and the perceived importance of garbage sorting (*β* = 0.0598, *p* = 0.0012, CI = [0.0237, 0.0959]). However, objective knowledge is not found to associate with voluntary behavioral intention (*β* = 0.0343, *p* = 0.1330, CI = [−0.0105, 0.0791]) on the 0.05 significance level. Next, subjective knowledge positively predicts the perceived importance of garbage sorting (*β* = 0.2289, *p* < 0.0001, 95% CI = [0.1417, 0.3161]) and voluntary behavioral intention (*β* = 0.2843, *p* < 0.0001, 95% CI = [0.1746, 0.3939]). Finally, perceived importance and voluntary behavioral intention are found to have a positive association (*β* = 0.2631, *p* < 0.0001, 95% CI = [0.1643, 0.3619]). Therefore, H2, H4, H6a, H6b, and H7b are supported, and H7a is not supported.

**Figure 2:**
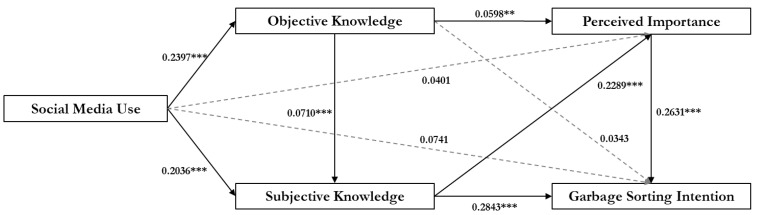
Sequential mediation model with coefficients. Note. ** *p* < 0.01, *** *p* < 0.001.

The coefficients of each path are presented in Figure 2.

Despite the above results, this study aims to further investigate the mediation effects of the variables. Following the suggestion of Hayes and Rockwood, it is not sufficient to use the *p*-value of the regression model to judge mediation effects, which should be treated as wholes and judged using confidential intervals [47]. Therefore, this study used the bias-corrected bootstrapping method to verify the mediation effects. The results of direct and indirect effects are presented in Table 3 and Table 4.

As shown in Table 3, the total effect of social media use on voluntary garbage sorting intention is positive (Effect size = 0.1726, 95% CI = [0.0938, 0.2514], the confidential interval does not contain 0), while the direct effect of social media use on voluntary garbage sorting intention is not significant (Effect size = 0.0741, 95% CI = [−0.0041, 0.1523], the confidential interval contains 0).

As for the indirect effects, the total indirect effect is found to be positive (Effect size = 0.0985, 95% CI = [0.0554, 0.1475], the confidential interval does not contain 0). As for the potential indirect effects, the mediation effect of objective knowledge between social media use and garbage sorting intention (Effect size = 0.0082, 95% CI = [−0.0013, 0.0259], the confidential interval contained 0), and the mediation effect of perceived importance between social media use and garbage sorting intention (Effect size = 0.0105, 95% CI = [−0.0081, 0.0336], confidential interval contains 0), are not found. The details of all possible indirect effects are listed in Table 4.

## 5. Discussion

This study examines the association between social media use and voluntary garbage sorting intention among adolescents, especially the underlying mechanism. Overall, this study demonstrates that social media use is positively and indirectly associated with adolescents’ intention to sort garbage voluntarily. Specifically, the results show that social media use is positively related to both objective and subjective knowledge of garbage sorting. Objective knowledge is positively related to subjective knowledge and perceived importance, while subjective knowledge is positively related to perceived importance and garbage sorting intention. Meanwhile, perceived importance has a positive relationship with behavioral intention. 

First, in line with the results of Ho et al.’s study [33], we found that social media use positively predicts adolescents’ objective and subjective knowledge. However, our results fail to demonstrate the direct effect of social media use on perceived importance and behavioral intention, which indicates that the impact of social media use on voluntary garbage sorting intention is mediated by knowledge. A closer look at the findings revealed that there exists a nuanced difference between objective knowledge and subjective knowledge regarding their mediation effects. To be specific, objective knowledge alone does not mediate the relationship between social media use and intention, and it only functions as one of the mediators in combination with the other variables, such as perceived importance. By contrast, the relationship between social media use and behavioral intention is mediated by either subjective knowledge alone or the conjoined function of subjective knowledge and perceived importance. These results not only highlight the importance of knowledge in the effect of social media use on adolescents’ intention towards garbage sorting, but also helps us to better understand the different mediating roles of objective and subjective knowledge in linking social media use and garbage sorting intention.

Next, our research results also indicate that both objective and subjective knowledge are positively associated with perceived importance and garbage sorting intention, which is somewhat different from the findings of Ho et al. [33]. In their research, Ho et al. found that individuals’ objective knowledge positively predicted their support for the policy of science and technology development, while subjective knowledge was negatively related to it [33]. This inconsistency probably results from the distinct dependent variables of these two studies. Specifically, the study of Ho et al. investigated people’s support for the governmental policy for science and technology as a dependent variable, and this construct was measured as the extent to which respondents agree or disagree with the statement that the government should fund research and scholars or not, whereas our study used individual behaviors or behavioral intention as the variable of interest. It is understandable that although a person shows positive attitude towards a policy, he may not perform what the policy requires due to other factors. Therefore, future research is needed to identify what impacts objective and subjective knowledge have on individuals’ ensuing attitude and behaviors.

Another finding worth discussing is the significant contribution of the perceived importance of garbage sorting in predicting people’s behavioral intention. While past studies have detected many antecedents of pro-environmental behavior, including external factors (such as socioeconomics, social, and cultural factors) and internal factors (such as motivation, environmental knowledge, awareness, and values and attitudes) [38,48], fairly little attention has been given to the construct of perceived importance. This study incorporated perceived importance into the conceptual model and confirmed that it not only directly predicts adolescents’ garbage sorting intention, but also functions as a mediator between knowledge and behavior intention. 

## 6. Conclusions

From the point of theoretical contribution, this study adds to the body of existing knowledge on the relationship between media use and pro-environmental behavior by examining how social media use impacts individuals’ voluntary garbage sorting intention in adolescents. While previous studies have studied the effects of media on pro-environmental behavior, few have examined the effects of social media use, particularly those on adolescents’ garbage sorting behavior. Beyond that, this study uncovers the underlying mechanism that social media use influences adolescents’ voluntary intention to sort garbage through the mediation of objective knowledge, subjective knowledge, and perceived importance. Such a research endeavor helps us to gain a deeper understanding of the way how social media use affects individuals’ pro-environmental behaviors. 

Additionally, this study has some practical implications regarding how to improve adolescents’ intention to sort garbage voluntarily. First of all, understanding the important role social media use plays in predicting adolescents’ garbage sorting intention, those involved in protecting the environment (including the government and NGOs) need to post more attractive environmental content on social media and encourage adolescents to actively engage in online discussion with others as well. In addition, we also found the dynamic relationship between the two types of knowledge that objective knowledge can mediate the relationship between social media use and intention only when it is transformed into subjective knowledge (or perceived importance). Since the possession of objective knowledge is not always equal to subjective knowledge and perceived importance [49], when harnessing social media to encourage adolescents to participate in sorting garbage, practitioners should frame and convey the messages to not only increase individuals’ actual possession of knowledge possession, but also to improve their subjective knowledge and perceived importance of the issue. For instance, they should provide interesting stories to attract the public’s attention. As such, adolescents will be more interested in consuming the information about garbage sorting, and thus perceive they are gaining related environmental knowledge. Additionally, the terrible outcomes of improperly disposing of garbage can be woven into those stories to enhance the adolescents’ perceived importance of the subject.

Despite the above contribution, this study has several limitations. Firstly, this study collected data from middle schools in Shanghai, the first pilot city to introduce a garbage sorting policy. To improve the generalizability of the results, future studies should collect samples not only from cities which have implemented a garbage sorting policy, but also from areas where this policy has not been implemented. Secondly, the data of this study is cross-sectional, thereby making causal inferences impossible to establish. Research designs as longitudinal surveys or field experiments can be adopted to compensate for this limitation in the future. Finally, this study touches on the two main functions of social media (i.e., information gaining and interpersonal communication). However, social media use behavior is complex, and different behaviors might have varying effects [50]. As such, a more comprehensive measurement of social media use should be employed to offer more insights on the question under discussion.

## Figures and Tables

**Table 1:** Descriptive statistics and correlation coefficients of key variables.

	M	SD	SMU	OK	SK	PI	GSI
**SMU**	2.691	0.977	1				
**OK**	7.339	1.681	0.159 ***	1			
**SK**	3.862	0.711	0.277 ***	0.179 ***	1		
**PI**	4.571	0.757	0.125 **	0.162 ***	0.264 ***	1	
**G** **SI**	3.899	0.964	0.183 ***	0.171 ***	0.285 ***	0.272 ***	1

Note. ** *p* < 0.01, *** *p* < 0.001; M, mean; SD, standard deviation; SMU, social media use; OK, objective knowledge; SK, subjective knowledge; PI, perceived importance; GSI, garbage sorting intention.

**Table 2:** Regression analysis.

Dependent Variable	OK		SK		PI		GSI	
	Coef.	SE	Coef.	SE	Coef.	SE	Coef.	SE
Independent variables								
SMU	0.2397 ***	0.0693	0.2036 ***	0.0286	0.0401	0.0323	0.0741	0.0398
OK			0.0710 ***	0.0167	0.0598 **	0.0184	0.0343	0.0228
SK					0.2289 ***	0.0444	0.2843 ***	0.0558
PI							0.2631 ***	0.0503
Control variables								
gender (male = 1)	−0.0844	0.1350	0.1037	0.0552	−0.0161	0.0601	−0.0463	0.0740
age	0.1446 ***	0.0338	−0.0630 ***	0.0140	−0.0377 *	0.0155	0.0555 *	0.0191
parent’s education	0.0772 *	0.0335	0.0123	0.0137	0.0023	0.0149	0.0271	0.0184
constant	3.4844 ***	0.7041	3.4514 ***	0.2933	3.6477 ***	0.3537	−0.0396	0.4721
Model fit								
*R*	0.2546		0.3603		0.3072		0.3952	
*R* ^2^	0.0648		0.1298		0.0944		0.1562	
*F*	10.3968 ***		17.8677 ***		10.3877 ***		15.7842 ***	

Note. * *p* < 0.05, ** *p* < 0.01, *** *p* < 0.001; OK, objective knowledge; SK, subjective knowledge; PI, perceived importance; GSI, garbage sorting intention; SMU, social media use; coef., coefficients; SE, standard error.

**Table 3:** Total and direct effects and the confidential intervals.

	Effect	SE	LLCI	ULCI
Total effect	0.1726	0.0401	0.0938	0.2514
Direct effect	0.0741	0.0398	−0.0041	0.1523

Note. SE, standard error; LLCI, lower-level confidence intervals; ULCI, upper-level confidence intervals.

**Table 4:** Indirect effects and the bootstrapping confidential intervals.

Indirect Effect	Effect	Boot SE	BootLLCI	BootULCI
Total	0.0985	0.0235	0.0554	0.1475
SMU → OK → GSI	0.0082	0.0067	−0.0013	0.0259
SMU → OK → SK → GSI	0.0048	0.0030	0.0013	0.0139
SMU → OK → PI → GSI	0.0038	0.0026	0.0007	0.0112
SMU → OK → SK → PI → GSI	0.0010	0.0008	0.0002	0.0034
SMU → SK → GSI	0.0579	0.0175	0.0290	0.0985
SMU → SK → PI → GSI	0.0123	0.0047	0.0052	0.0245
SMU → PI → GSI	0.0105	0.0104	−0.0081	0.0336

Note. SMU, social media use; OK, objective knowledge; SK, subjective knowledge; PI, perceived importance; GSI, garbage sorting intention; Boot SE, bootstrapped standard error; Boot LLCI, bootstrapped lower-level confidence intervals; Boot ULCI, bootstrapped upper-level confidence intervals.

## Data Availability

The data presented in this study are openly available contacting corresponding Author.
